# Contrast‐Enhanced Computed Tomography (CT) With Concordant Sonography as Sufficient Early Detection Tools for Recurrent and Persistent Cervical Metastases After (Chemo)radiotherapy (CRT)

**DOI:** 10.1002/hed.28008

**Published:** 2024-11-19

**Authors:** Carl Stöcker, Jens Greve, Meinrad Beer, Beate Hosch, Thomas F. E. Barth, Thomas K. Hoffmann, Adrian von Witzleben

**Affiliations:** ^1^ Department of Otorhinolaryngology, Head & Neck Surgery University Hospital Ulm Ulm Germany; ^2^ Department of Diagnostic and Interventional Radiology University Hospital Ulm Ulm Germany; ^3^ Department of Radiotherapy University Hospital of Ulm Ulm Germany; ^4^ Department of Pathology University Hospital of Ulm Ulm Germany

**Keywords:** computed tomography, HNSCC, neck dissection, recurrence, sonography

## Abstract

**Background:**

Most challenging treatment needs are in recurrent or persisting head and neck squamous cell carcinoma (HNSCC) patients after (((chemo‐)radiotherapy) (C)RT).

**Materials and Methods:**

This 10‐year retrospective study included 100 patients, who initially received (C)RT followed by neck dissection (ND). The results of computed tomography (CT) and sonography were evaluated for residual/recurrent cervical lymph nodes and compared to the histopathology. On this basis we calculate the sensitivity, specificity, negative predictive value (NPV) and positive predictive value (PPV).

**Results:**

A total of 144 ND specimens were analyzed. The combination of CT and sonography (*n* = 103) reached values 97% sensitivity, 71% specificity, 98% NPV, 66% PPV, and 81% overall accuracy.

For patients who received as primary treatment CRT the values for the combined imaging were: 100.0%, 73.5%, 100.0%, 66.7% and 82.7% respectively.

**Conclusion:**

Our study demonstrates that the combined use of CT and sonography reliably detects lymph node metastases, particularly in patients previously treated with CRT, even after a long time after treatment.

## 1 Introduction

One of the most crucial factors that significantly influences survival is the involvement of local lymph nodes. When these are affected, the survival prognosis can decrease by 50% [1].

Except for early‐stage oral cavity cancers, managed with surgery alone, and larynx cancers, which can be addressed through surgery or radiation alone, the treatment of most head and neck squamous cell carcinoma (HNSCC) cases necessitates multimodal approaches. Consequently, a multidisciplinary care approach is essential: Locoregionally advanced HNSCC is managed through either a combination of surgery followed by risk‐adapted adjuvant ((chemo‐)radiotherapy) ((C)RT) or definitive CRT [2]. Owing to the significant influence on overall survival and locoregional control, the treatment of local lymph nodes necessitates a well‐established and robust therapeutic approach.

Recurrent or persistent tumor or metastatic states following completed (C)RT represent one of the most complex clinical scenarios for HNSCC patients [3]. Post‐primary therapy, there are various clinical scenarios that elucidate this status. Fundamentally, the recurrence rate of the tumor itself depends on the location and stage of the disease. Over a period of 5 years, less than 50% of patients remain progression‐free [4]. Subsequent therapy typically involves surgical salvage procedures, as they significantly improve survival in comparison to non‐surgical therapy [5]. However, irrespective of the primary tumor, the presence of occult lymph node metastases in cN0 cases should not be overlooked. The incidence of these metastases varies depending on the primary tumor site, ranging from 12.9% for oral cavity tumors to 27.3% for hypopharyngeal tumors [3]. The average rate of occult metastases for patients with advanced HNSCC lies at approximately 15.4% [3].

On the other hand, the group led by Mehanna et al. demonstrated that, fundamentally, in the cohort of patients immediately following CRT, there was no significant difference in survival outcomes among patients with advanced lymph node stages (N2, N3), when comparing post‐therapeutic observation using PET‐CT (positron emission tomography‐computed tomography) with planned neck dissection (ND) [6].

In cases of newly emerging recurrence following complete remission, which may occur at any given time [7] but is particularly frequent within the first 2 years [8], earlier presentation is always possible. Consequently, continuous post‐therapeutic follow‐up is conducted, consisting of clinical examinations and ultrasonography at three‐month intervals up to the 30th post‐therapeutic month, followed by six‐month intervals until the fifth post‐therapeutic year.

Within the cross‐sectional imaging diagnostics, initially conducted 3 months post‐therapeutically to establish a post‐treatment reference point and subsequently performed annually, radiologists and nuclear medicine specialists face a challenging situation. This challenge is compounded in patients who have undergone RT (radiotherapy), due to the thickening of the skin, platysma, pharyngeal walls, and laryngeal structures. Furthermore, in addition to the atrophy of the lymphatic tissue, including the Waldeyer's pharyngeal ring and cervical lymph nodes, edema in the retropharyngeal space can also be observed [9]. Moreover, one‐year post‐radiation, 58% of patients exhibited fibrosis in the irradiated area [10]. This fibrosis also contributes to the difficulty in evaluating lymph node metastases, especially early post‐CRT [11, 12].

Irrespective of the previously described scenarios where patients are subjected to any form of post‐therapeutic persistent or recurrent tumor diseases, imaging diagnostics of these patients play a crucial role in offering the best possible therapy. It has been demonstrated that PET‐CT imaging can prevent overtreatment, in terms of elective post‐therapeutic NDs [6]. The current focus is to determine how more accessible, cost‐effective, and simpler imaging techniques can support these findings even in patients who exhibit indications of tumor recurrence many years post‐therapy.

Therefore, the primary objective of this study was to retrospectively demonstrate how computed tomography (CT) and sonography (yNx), utilized during guideline‐restaging after prior (C)RT of the lymph nodes, were able to accurately represent the pathological actual lymph node status (pNx). Our research question investigates patients who underwent only (C)RT as their primary therapeutic intervention.

## 2 Methods

**FIGURE 1:**
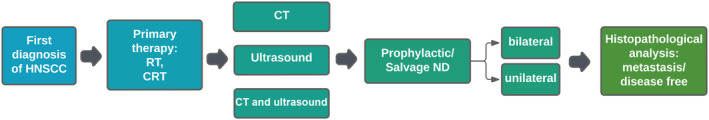
Patient cohort in flow chart. Abbreviations: CRT, Chemoradiotherapy; CT, Computed tomography; HNSCC, Head and neck squamous cell carcinoma; ND, Neck dissection; RT, Radiotherapy. [Color figure can be viewed at wileyonlinelibrary.com]

This retrospective study encompassed HNSCC patients from the Department of Otolaryngology and Head & Neck Surgery at our Hospital. Following the approval granted by the Ulm Ethics Committee (No. 138/22), patients were identified through the Comprehensive Cancer Center Ulm (CCCU) tumor registry. The selection of relevant patients was then successively refined within the CCCU, as detailed in the provided Figure 1. Additionally, a digital search within the department‐specific patient management software, ePA, using the term “Salvage Neck Dissection” was conducted.

### 2.1 Participants

All the patients included in the study initially presented with a pathologically confirmed HNSCC and received as a primary therapy (C)RT of the cervical lymph nodes and the primary tumor. Within the national comprehensive cancer network (NCCN) guideline‐conformant restaging or any subsequent time the patients presented with either a recurrence or persisting primary tumor or recurrent or persistent local metastasis. These patients underwent either prophylactic ND due to persistent or recurring primary tumors, according to guideline‐recommended treatment based on the localization and extent of the persistent/recurrent tumor. Additionally, salvage ND was performed when imaging indicated persisting or recurring lymph node metastasis. By comparing the preoperative imaging data with the final histopathological outcomes, metrics such as sensitivity, specificity, negative predictive value (NPV) and positive predictive value (PPV) were determined.

The preoperative imaging was primarily conducted and discussed by board‐certified radiologists from our Hospital for CT scans. A smaller fraction (< 10%) were performed externally. The criteria used to distinguish malignant from benign lesions using contrast‐enhanced CT scans was based on standard widely known criteria of malignancy, such as size (> 10 mm), shape (round to oval) and perfusion as well as the comparison to the pre‐therapeutical imaging as reference. In the case of sonography, trained otolaryngologists carried out the procedures. The evaluation of suspicious lymph nodes included criteria such as size, shape, perfusion, as well as factors like the presence of a hilum sign. Importantly, every suspicious lymph node underwent cross‐sectional imaging to confirm suspected lesions, as these findings alone do not solely guide therapeutic decisions.

Instead, decisions for or against a ND were not solely based on imaging results. In our clinic decisions regarding treatment are made by an interdisciplinary tumor board, which includes ENT specialists, radiologists, nuclear medicine specialists, pathologists, radiation therapists, and occasionally maxillofacial surgeons. Here, the imaging results are combined with the preoperative biopsy and clinical findings, the patient's overall condition, the boundaries of prior radiation therapy and their personal preferences. Treatment decisions are made based on this comprehensive assessment, ensuring a patient‐tailored approach.

All ND specimens were postoperatively diagnosed at our Institute of Pathology. Although each individual lymph node was pathologically examined, for simplicity, only the hemi necks were included in the analysis. A random sampling verification was conducted, focusing exclusively on lymph nodes within specific neck levels, to confirm that they corresponded to those described in the pathology reports.

#### 2.1.1 Data Analysis

From January 2010 to May 2022, a retrospective study that included 100 patients was conducted. These patients were analyzed on both neck sides.

Through radiological findings, specifically selected for each patient on a site‐specific basis, these results could be correlated with and compared to postoperative histopathological findings. Similarly, the institute's internal sonographic findings of the patients were also utilized for the same comparative analysis.

Lymph node metastases detected via the aforementioned imaging modalities were categorized as true‐positive when their existence was substantiated by histopathological evaluation. Instances of an overestimated pathological classification (pN) led to a false‐positive categorization. Conversely, lymph node metastases were classified as false‐negative when histopathological examination affirmed their presence, yet they remained elusive to the imaging techniques. In scenarios where the absence of metastatic disease was accurately identified, patients were designated as true‐negative.

For each imaging modality, the metrics of sensitivity, specificity, accuracy, PPV and NPV, were calculated, focusing on two primary objectives: accurate neck staging and the distinction between N0 and N+ classifications.

In addition to the previously described analyses conducted on all patients, another assessment was exclusively performed for the subgroup of patients who received CRT. For this specific cohort, the parameters were recalculated, providing a focused evaluation of the diagnostic efficacy of imaging in the context of CRT.

## 3 Results

### 3.1 Demographics

Table 1 details the demographic and clinical characteristics of the patient cohort (*n* = 100) in this study, revealing a mean age of 62.8 years and a median of 63.5 years, with an age range from 19 to 86 years. The cohort primarily comprised patients with oropharyngeal carcinomas, representing 38% of the total, while hypopharyngeal and laryngeal cancers each accounted for just over one‐fifth of cases. A majority of the patient cohort, constituting 82%, were male.

**TABLE 1:** Clinical characteristics of the patient cohort before salvage or prophylactic ND.

Charateristics	Value (*n*)
Age (median)	62.8 (63.5) years
Sex	
Male	82
Female	18
Smoking	
Smokers	57 (39,2 pck yrs)
Smoking history	19 (37,4 pck yrs)
non‐smokers	20
not determined	4
Primary site	
Oropharynx	38
Hypopharynx	23
Larynx	22
Nasopharynx	7
Oral cavity	1
Others	9
Primary stage	
yT_x_	6
yT_0_	13
yT_1_	12
yT_2_	11
yT_3_	23
yT_4_	35
Neck stage	
yN_x_	4
yN_0_	22
yN_1_	14
yN_2_	48
yN_3_	12
Distant metastasis	
yM_x_	14
yM_0_	84
yM_1_	2
Pathological findings	
Histopathological positive findings	48
Medium diameter of metastasis‐positive lymph nodes	14.2 mm
Medium diameter of metastasis‐negative lymph nodes with suspected metastasis	18.2 mm
HPV positive patients without smoking history	4 (Oropharyngeal)
HPV positive patients with smoking history	5 (4 oropharyngeal)
Number of micrometastases	11 (22.9% of all metastases)

Abbreviations: pck yrs, Pack years; yN, Clinical distant metastasis stage after primary treatment; yN, Clinical lymph nodal stage after primary treatment; YT, Clinical tumorstage after primary treatment.

The known etiological factors contributing to the development of persistent or recurrent tumor diseases, such as tobacco abuse, accounted for 76% of patients in this study. Patients who persisted in smoking before ND had an average of 39 pack‐years, while those with a history of tobacco abuse averaged 37 pack‐years. Out of the 100 patients, 9 presented with an HPV‐positive tumor, all of which could be attributed to the oropharynx. Among these, three patients exhibited persistent tobacco abuse, and two were former smokers.

The study involved a structured analysis of various lymph node therapies, as delineated in Figure 1 and for each patient discussed prior within the tumor board. Treatment modalities included RT for 46 patients, primarily targeting local lymph nodes, and CRT for an additional 54 patients. These interventions were subject to reevaluation via imaging at different time point. In the average, the timing of imaging occurred at 332 days following the completion of primary therapy, with the earliest patient requiring ND 21 days after primary therapy completion, and the latest patient becoming treatment‐dependent nearly 7 years after completion of (C)RT.

Post‐tumor board imaging results facilitated stratification of patients into distinct therapeutic groups.

1. The first group, consisting of 24 patients, underwent prophylactic ND in the absence of imaging or clinical indicators of lymph node metastasis due to a salvage operation of the primary, yielding 45 histopathological specimens.
2. The salvage ND group encompassed patients with suspected lymph node involvement, independent of primary tumor status, comprising 60 patients with 73 ND histopathological specimens from both unilateral and bilateral treatments.
3. The third, combined group involved 13 patients who received on one side prophylactic ND and salvage ND on the other, resulting in 26 specimens.

Preoperative assessment revealed a predominance of advanced‐stage primary tumors, with nearly 60% of patients presenting with either T3 or T4 tumors. The most frequent post‐therapeutic nodal stage was N2, observed in 48 patients, while 22 patients were categorized as N0. Further classification details and staging information are available in Table 1.

Out of the 144 histopathological specimens provided, 48 exhibited metastases. The average diameter of specimens with metastasis was 14.2 mm, whereas nontumorous inflamed lymph nodes showed an average diameter of 18.2 mm.

### 3.2 Imaging and Pathology Results at the Time Point Before Salvage or Prophylactic ND

Table 2 provides a comparative analysis of the preoperative imaging results with the subsequent pathological findings. The derived metrics, as detailed below, were achieved based on the comparison of imaging and histopathological results.

**TABLE 2:** Imaging results with merge of preoperative imaging techniques and postoperative histopathological findings.

Bildgebung	Heminecks (*n*)	Sensitivity	Specificity	NPV in %	PPV in %	Accuracy
CT	120	95.4	64.6	96.1	59.4	75.0
Sonography	143	90.0	66.7	92.5	59.2	74.8
Combination: CT + sonography	103	97.4	70.8	97.9	66.1	80.6
Combination: CT + sonography (CRT)	34	100.0	73.5	100.0	66.7	82.7

In this study, CT was conducted on 120 heminecks. Of the pathologically identified metastases, 95.4% were accurately detected by CT, resulting therefore in a NPV of 96.1%. Additionally, CT was able to correctly identify approximately 65% of pathologically negative neck nodes, yielding a PPV of 59.4%.

The diagnostic accuracy was further enhanced by incorporating sonography. When results from both modalities were concordant, sensitivity increased to 97.4%, specificity to 70.8%, NPV to nearly 98%, and PPV to 66.0%.

Standalone sonography, carried out on 143 heminecks, demonstrated a sensitivity of 90%, specificity of 66.7%, NPV of 92.5%, and a PPV of 59.2%. Interestingly, the direct comparison of standalone sonography and standalone CT showed no statistically different results, which underpins the importance of the sonography.

Beyond the initial analysis of all imaging data, a detailed examination specifically targeting the subgroup of patients who received prior CRT is warranted to ascertain sensitivity, specificity, NPV and PPV. In this specific patient subset, combination imaging accurately identified 18 lymph node metastases as positive, with no recorded false‐negative outcomes. This translates to a sensitivity of 100.0%. When evaluating specificity, we found 9 false‐positive imaging results and 25 accurate negative findings, resulting in a specificity of 73.5%. Based on these metrics, the derived NPV stands at 100.0% and the PPV at 66.7%.

## 4 Discussion

The primary objective of this study was to evaluate the efficacy of CT and sonography. For this purpose, the diagnostic accuracy of both individual and combined imaging was assessed, as presented in Table 2. The findings suggest that the combination of CT and sonography exhibited a nominal superiority in terms of sensitivity and specificity.

This combination, irrespective of the initial treatment modality, achieved a sensitivity of 97.4%, a specificity of 70.8%, resulting in a NPV 97.9% and a PPV of 66.7%.

Globally, the quality of various imaging modalities has been extensively researched. In this context, a study conducted by Liauw et al. which compared the quality of CT images, demonstrated a sensitivity of 96% across 266 images [13]. The most pertinent study related to this patient cohort utilizing CT imaging revealed a specificity of 76% [14].

Employing sonography, sensitivity values ranged between 78% [15] and 92% [16], whereas the specificity values fluctuated between 28% [16] and a maximum of 79.4% [17].

After extensive evaluation of the combined advantages of CT and sonography, a pivotal question arises: Does this combined imaging approach offer a feasible alternative to the current guideline‐recommended prophylactic ND for patients presenting with recurrent or persistent primary tumors, but with no evident lymph node changes post‐CRT? emphasis was primarily placed on patients with negative imaging results, while those with positive preoperative imaging outcomes will be discussed separately in a subsequent section.

International studies, such as Brkovic et al. focused on patients initially manifesting lymph node metastases but later showed unremarkable lymph nodes post‐therapy via PET‐CT and CT [18]. While the study illuminated a notably high NPV influenced by a single false‐negative lymph node, definitive outcomes remained elusive. Brkovic's team suggested sparing patients with initial N1 tumors displaying complete remission on CT from further surgery [18].

The team of Pellini et al. documented high NPV values combining PET‐CT and sonography, which supported the potential feasibility of post‐CRT monitoring without additional ND [15]. This is further endorsed by the generally low recurrence rates post‐CRT, ranging between 2.3% [19] and 10.3% [20]. Another noteworthy observation is that in the event of lymph node metastasis recurrence, it predominantly occurs at the same level as the original metastasis and makes therefore extended ND redundant [15].

Our study also underscored high sensitivity and NPV values, reaching 100% sensitivity for patients previously treated with CRT. Preliminary data suggest that modifying current clinical approaches might reduce postoperative complication rates, thereby potentially enhancing patients' post‐therapeutic quality of life.

Given the intrinsic low NPV due to the minor prevalence rate of lymph node recurrence [21, 22, 23], it is evident that such recurrences are infrequent, regardless of imaging quality. While arguments support imaging monitoring post‐initial CRT, potential non‐surgical monitoring risks must be addressed.

In 2016, it was demonstrated in a multicenter study that patients undergoing continuous post‐therapeutic PET‐CT monitoring did not exhibit any survival disadvantage when compared to the group receiving planned ND both pre and post‐therapy [6]. This study enrolled patients with extensive tumor and nodal stages, all of whom received CRT as primary treatment [6]. Furthermore, our study revealed that this outcome could be achieved also through the capabilities of readily accessible and cost‐effective imaging modalities, such as CT and ultrasound. Therefore, following a certain reconfirmation process, consideration could be given to omit planned post‐CRT ND, even with the availability of affordable imaging techniques.

On the other hand, data regarding occult micrometastases in clinically inconspicuous lymph nodes should not be disregarded, and they should be duly considered based on the location of the primary tumor [3].

Outside conventional and metabolic imaging modalities, sonography was explored for its efficacy. Yom et al.'s analysis supports delaying or even omitting prophylactic ND in cases of unremarkable lymph node findings [16]. Conventional imaging techniques often face diagnostic challenges within the first 3 months post‐therapy, with sonography potentially offering valuable complementary insights [24, 25]. It is noteworthy to mention that, in comparison to all cross‐sectional imaging techniques, ultrasonography is more accessible and significantly more cost‐efficient [26].

Based on our examination of 143 neck sides, we identified a sensitivity of about 90% and nearly 67% specificity. Although these are no peak values, this imaging technique primarily serves as a supplementary tool, providing significant insights when combined with CT imaging. It is crucial to acknowledge institutional differences in terms of resource availability and expertise [26].

Our retrospective study remains inconclusive in advocating against prophylactic ND. Radiomics represents a promising avenue for future research in the diagnosis of regional lymph node metastases. Previous studies have demonstrated that radiomics models, when combined with clinical examination, can outperform conventional methods in diagnosing lymph node metastases in oral cavity carcinomas. However, no significant improvement in sensitivity compared to traditional imaging techniques has been observed to date [27]. However, this study has demonstrated that in clinical practice, over the past several years, promising and satisfactory results have already been achieved using the combination of CT and ultrasound. Therefore, in addition to PET‐CT, more cost‐effective and readily available methods have shown good outcomes as well.

The diminished specificity observed in our study, which has been documented in numerous international investigations [15, 16, 18, 28, 29, 30], necessitates rigorous deliberation regarding the etiology of such findings and potential preventive strategies. Multiple factors might contribute to false‐positive outcomes. Notably, the timing of the imaging is pivotal. It has been established that imaging conducted 4 weeks post primary therapy does not provide reliable insights regarding the long‐term success of the locoregional condition [31], associated with postoperative hypermetabolic activity. Additionally, post‐radiative inflammatory reactions and potential residual disease manifestations, which may resolve over time due to radiation aftereffects, should be considered [32, 33]. Haerle et al. showed that the absence of contrast agents following tumor‐associated necroses results in increased perilymphatic degradation reactions linked to inflammation, which led to a surge in false‐positive findings [26, 34, 35, 36].

Consequently, imaging should be conducted 8–12 weeks following the definitive therapy. Adhering to this timeframe is crucial to not exceed the optimal surgical intervention period, where is still moderate fibrosis present. However, it is important to consider that recurrent tumors may manifest at any time, even years after the completion of primary therapy, which might justify the use of imaging modalities at various other time points. Additionally, patient compliance should not be overlooked, as not all patients adhere to regular follow‐up appointments, potentially leading to deviations in the timing of these imaging sessions.

Considering all the previously mentioned improvements in imaging techniques, when imaging indicates a positive result, patients should be treated in accordance with their suspected condition, for example metastasis. This means that, all patients presenting with suspicious lymph node findings should undergo salvage ND. Without intervention, survival rates for patients with hypopharynx carcinomas were observed to be around 20% after 12 months [37] as current imaging techniques do not entirely depict the intricacy of head–neck tumors and their metastasis.

## 5 Conclusion

In summary, we demonstrate that the combination of CT and ultrasound can yield reliable results when findings are concordant. This is particularly applicable to patients who have previously received CRT as their primary therapy.

Regardless of the timing of imaging, the aforementioned combination achieves high detection rates of lymph node metastases, even many months or years after treatment. Considering the ease of availability and cost‐effectiveness in comparison to PET‐CT, this is an important finding that may be of interest for future studies. The low specificity rates for CT and Sonography described by many authors were also confirmed in our study and can only be improved through methodological enhancements.

## Data Availability

The data can be made available upon request from the authors.
